# Validation of Clinical Characteristics and Effectiveness of Pulmonary Rehabilitation in a COPD Population with Discrepancy between Exercise Tolerance and FEV1

**DOI:** 10.3390/healthcare9010053

**Published:** 2021-01-06

**Authors:** Jun Horie, Koichiro Takahashi, Shuuichi Shiranita, Kunihiko Anami, Shinichiro Hayashi

**Affiliations:** 1 Department of Physical Therapy, Faculty of Health Science, Kyoto Tachibana University, 34 Yamada-cho Oyake, Yamashina-ku, Kyoto 607-8175, Japan; 2 Division of Hematology, Respiratory Medicine and Oncology, Department of Internal Medicine, Faculty of Medicine, Saga University, 5-1-1 Nabeshima, Saga 840-8502, Japan; takahak@cc.saga-u.ac.jp; 3 Choseido-Watanabe Clinic, Department of Rehabilitation, 7182-58 Kaigandoori, Karatsu City, Saga 847-0873, Japan; choseido-reha@po.people-i.ne.jp; 4 Department of Rehabilitation, Faculty of Health Sciences, Naragakuen University, 3-12-1 Sangou-cho Tatsunokita, Ikoma-gun, Nara 636-8503, Japan; anami@naragakuen-u.jp; 5 Department of Physical Therapy, School of Health Sciences at Fukuoka, Okawa Campus in Fukuoka Prefecture, International University of Health and Welfare, Okawa, 137-1 Enotsu, Okawa City, Fukuoka 831-8501, Japan; hayashs@hotmail.co.jp

**Keywords:** chronic obstructive pulmonary disease, COPD management, exercise tolerance, forced expiratory volume, rehabilitation

## Abstract

This study’s objective was to examine the characteristics of patients with chronic obstructive pulmonary disease (COPD) presenting with various exercise tolerance levels. A total of 235 patients with stable COPD were classified into 4 groups: (1) LoFlo + HiEx—patients with a six-minute walking distance (6MWD) ≥350 m and percentage of predicted forced expiratory volume in 1 s (%FEV_1.0_) <50%; (2) HiFlo + HiEx—patients with a 6MWD ≥350 m and a %FEV_1.0_ ≥50%; (3) LoFlo + LoEx—patients with a 6MWD < 350 m and %FEV_1.0_ < 50%; and (4) HiFlo + LoEx—patients with a 6MWD <350 m and %FEV_1.0_ ≥ 50%. Aspects of physical ability in the HiFlo + LoEx group were significantly lower than those in the HiFlo + HiEx group. The HiFlo + LoEx group was characterized by a history of hospitalization for respiratory illness within the past year, treatment with at-home oxygen therapy, and lacking daily exercise habits. Following three months of pulmonary rehabilitation, the LoFlo + HiEx group significantly improved in the modified Medical Research Council dyspnea score, maximum gait speed, and 6MWD, while the HiFlo + LoEx group significantly improved in the percentage of maximal expiratory pressure, maximum gait speed, 6MWD, incremental shuttle walking distance, and St. George’s Respiratory Questionnaire score. The HiFlo + LoEx group had the greatest effect of three-month pulmonary rehabilitation compared to other groups.

## 1. Introduction

For patients with chronic obstructive pulmonary disease (COPD), the most troublesome symptom is dyspnea. Dyspnea leads to reduced exercise tolerance and creates a host of impairments for patients with COPD [1]. In these patients, dyspnea during exercise is caused primarily by dynamic hyperinflation (DHI) due to increased functional residual capacity and decreased inspiratory capacity, both of which are caused by airflow obstruction during respiration [2]. Thus, dyspnea-induced reduction in exercise tolerance in patients with COPD is closely related to respiratory function, particularly the reduction of forced expiratory volume in 1 s (FEV_1.0_), which represents the degree of airflow obstruction during respiration (indicated as FEV_1.0_ or as a FEV_1.0_%, the percentage of predicted FEV_1.0_ (%FEV_1.0_), and the Global Initiative for Chronic Obstructive Lung Disease (GOLD) disease stage classification) [3].

The relationships among severity of airflow obstruction, DHI, and exercise tolerance have been examined in several studies. Pinto-Plata et al. [4] reported that while patients in Global Initiative for Chronic Obstructive Lung Disease (GOLD) stage 1 with mild airflow obstruction did not have reduced exercise tolerance, those in GOLD stage 2 started to show a decline in exercise tolerance. O’Donnell et al. [5] and Vogiatzis et al. [6] reported that patients in the later GOLD stages are more susceptible to DHI. Furthermore, reduced physical activity has been reported in patients who are in a more advanced GOLD stage and have DHI [7,8].

However, in our clinical experience, some patients may have high exercise tolerance despite severe airflow obstruction and others may have low exercise tolerance despite mild airflow obstruction. We refer to such discrepancies as “COPD patients presenting various exercise tolerance”; we define the former group as “the low expiratory flow—high exercise capacity (LoFlo + HiEx) group” and the latter group as “the high expiratory flow—low exercise capacity (HiFlo + LoEx) group”.

To date, GOLD has also shown that the benefits of pulmonary rehabilitation for COPD patients are significant [9]. It has been reported that pulmonary rehabilitation is the most effective treatment strategy for improving shortness of breath, health, and exercise tolerance [10]. In addition, it has been reported that all COPD patients benefit from it, especially in moderate to severely ill patients (including those with chronic hypercapnicity) [11]. However, as mentioned above, it is often seen clinically. There have been no studies focusing on the characteristics the LoFlo + HiEx and HiFlo + LoEx groups nor the effects of rehabilitation. In addition, the effects of pulmonary rehabilitation on COPD patients with inconsistency between exercise tolerance and FEV_1.0_ have not been examined. Therefore, the objective of this study was to examine the characteristics of patients in LoFlo + HiEx and HiFlo + LoEx groups by cross-sectional analysis of respiratory function, physical function, physical ability, psychological function, and social background. Furthermore, the study aimed to analyze the effect of a three-month pulmonary rehabilitation on the same groups, to establish differences in response to rehabilitation among patients with COPD.

## 2. Materials and Methods

### 2.1. Study Design and Setting

This prospective cohort study included a cross-sectional analysis of data at the start of a three-month pulmonary rehabilitation program (baseline) and a longitudinal analysis of the effects of such pulmonary rehabilitation. The study was conducted in a hospital rehabilitation unit.

### 2.2. Subjects

The subjects comprised 235 patients with stable COPD (216 men, 19 women). The exclusion criteria were a history of respiratory illness other than COPD, serious internal complications, a painful condition that would impede gait, dementia, or unsuitability for inclusion in the opinion of a physician. The study protocol was approved by the Institutional Review Board at Kyoto Tachibana University, and all subjects provided verbal and written informed consent.

### 2.3. Group Assignment

**Figure 1:**
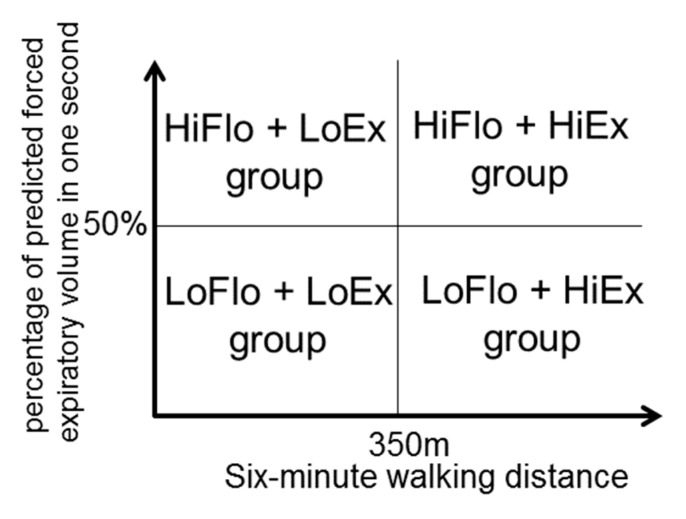
Classification of the LoFlo + HiEx, HiFlo + HiEx, LoFlo + LoEx, and HiFlo + LoEx groups.

To establish each participant’s exercise tolerance, the cut-off point for %FEV_1.0_ was 50%, which separates GOLD stages 1–2 from GOLD stages 3–4. To distinguish between high and low exercise tolerance among participants, the cut-off point for the six-minute walking distance (6MWD) was 350 m, which was considered “poor 6MWD” in the ECLIPSE study [12] and is used as a prognostic indicator in the BODE indices [13] (Figure 1).

Participants with a 6MWD ≥350 m and %FEV_1.0_ <50% were defined as the LoFlo + HiEx group, participants with 6MWD ≥350 m and %FEV_1.0_ ≥50% as the high expiratory flow—high exercise capacity (HiFlo + HiEx) group, patients with 6MWD <350 m and %FEV_1.0_ <50% as the low expiratory flow—low exercise capacity (LoFlo + LoEx) group, and patients with 6MWD <350 m and %FEV_1.0_ ≥50% as the HiFlo + LoEx group.

### 2.4. Measurement Indicators

The primary measurement indicators were 6MWD (used to distinguish between high and low exercise tolerance) and %FEV_1.0_ (used to establish exercise tolerance). Percentage of predicted forced vital capacity (%FVC) and respiratory muscle strength were used as indicators of respiratory function. Respiratory function was assessed using an Autospiro AS-507 (Minato Medical Science Co., Ltd., Osaka, Japan). More detailed investigations were performed in accordance with the American Thoracic Society’s/European Respiratory Society’s on the standardization of spirometry (2005) [14]. Respiratory muscle strength was assessed by measuring maximal expiratory pressure (MEP) and maximum inspiratory pressure (MIP) with the Autospiro AS-507. Both were measured twice, and the respective optimal values divided by body weight (%MEP and %MIP) were recorded.

The percentage of ideal body weight (%IBW), modified Medical Research Council dyspnea scale (mMRC) score, grip power, and ratio of knee extension strength to body weight (%KE) were used as indicators of physical function. The %IBW was defined as actual body weight divided by standard body weight (as calculated based on height). Grip strength was determined using Digital Grip Meter Grip D (Takei Scientific Instruments Co, Ltd., Niigata, Japan) by measuring the muscle strength during isometric contraction of the right and left hand (twice each), and the best values were considered as the measured values. Knee extension strength was assessed using a μTas M-1 hand-held dynamometer (Anima Corp., Tokyo, Japan). The maximum voluntary isometric contraction was measured twice in both knees (flexed at 90°); the optimal value divided by body weight was recorded.

Maximum gait speed (MGS), timed up and go time (TUG), 30-s chair stand times (CS-30), incremental shuttle walking distance (ISWD), activities of daily living (ADL), and health-related quality of life (QoL) were used as indicators of physical ability. ADL were assessed by the total score on the Nagasaki University Respiratory ADL questionnaire (NRADL). Health-related QoL was assessed by the St. George’s Respiratory Questionnaire (SGRQ). Psychological function was assessed using scores (including anxiety and depression subscales) on the Hospital Anxiety and Depression Scale (HADS).

Social background parameters included employment status, marital status, alcohol consumption, smoking, exercise habits, driving a car, comorbidities (cancer, heart disease, hyperlipidemia, hypertension, diabetes mellitus), use of home oxygen therapy, type of dwelling (one-story or two-story), environment around the home (flat or hilly), hospitalization for respiratory illness within the previous year, and acute exacerbation within the previous year.

### 2.5. Study Protocol

**Figure 2:**
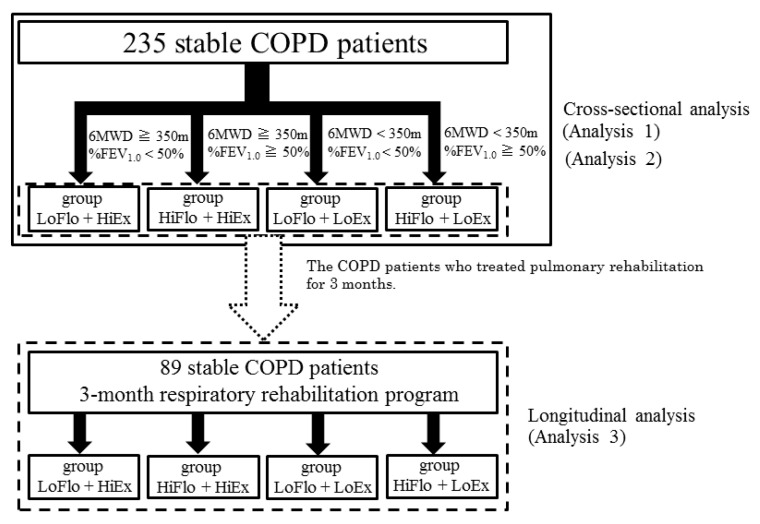
Study protocol. In total, 235 participants with stable COPD were assessed at baseline and divided into the LoFlo + HiEx, HiFlo + HiEx, LoFlo + LoEx, and HiFlo + LoEx groups. %FEV_1.0_, percentage of predicted forced expiratory volume in one second; 6MWD, six-minute walking distance.

A total of 235 participants with stable COPD were assessed at baseline and divided into the LoFlo + HiEx, HiFlo + HiEx, LoFlo + LoEx, and HiFlo + LoEx groups. The primary analysis of these patients consisted of cross-sectional assessment of indicators of respiratory function, physical function, and physical ability (analysis 1). A further cross-sectional analysis was performed to identify any relationships with social background (analysis 2). The patients then underwent pulmonary rehabilitation. Of the initial participants, 89 (79 men, 10 women) who were assessable at 3 months were used in a secondary longitudinal analysis of changes in indicators of respiratory function, physical function, and physical ability (analysis 3; Figure 2). No one was hospitalized or received additional treatment due to acute exacerbations during the period of respiratory rehabilitation.

The evaluators of the metrics in this study were the authors and their team of physiotherapists, and the respiratory rehabilitation program was implemented by physiotherapists who did not know the method of patient grouping in this study.

The basic respiratory rehabilitation program was implemented as an individual program, and the content included lower limb/trunk strength training (standing up movement, heel raising movement, table lifting movement) 10 times each × 3 sets, aerobic training (load intensity of about 50% of maximum exercise capacity calculated from ISWD at the time of initial evaluation, and the program was carried out using a bicycle ergometer) × 30 min, and breath training and limb stretching as other conditioning (preparatory exercise).

One session of the program lasted 60 min (including ADL instruction and advice) and was conducted twice a week. The implementation period was 90 days after completing the initial evaluation (25 sessions up to the evaluation after 3 months in all groups).

### 2.6. Statistical Analysis

Explanatory indicators of the LoFlo + HiEx, HiFlo + HiEx, LoFlo + LoEx, and HiFlo + LoEx groups at baseline were compared using one-way analysis of variance (ANOVA). The relationships between group classification and participants’ social background were analyzed using Pearson’s χ^2^ test; differences in frequencies were assessed based on adjusted residuals of ≥2 or <2.

Comparisons between the four groups and between baseline and post-pulmonary rehabilitation assessments were performed using ANOVA with a two-factor spilt-plot design. The changes occurring between baseline and after pulmonary rehabilitation were calculated using the following formula: (values measured after pulmonary rehabilitation−values measured at baseline)/values measured at baseline) ×100. Post hoc testing was performed using the Bonferroni procedure. A between-group comparison of changes between baseline and post-pulmonary rehabilitation was performed using a one-way ANOVA. The sample size required for one-way analysis of variance for a baseline group comparison, calculated using effect size (=0.25) and power of test (=0.8), the required sample size was equivalent to 180.

The level of statistical significance was set at 5%. The statistical analysis was undertaken using SPSS version 22.0 software (IBM Corp., Armonk, NY, USA).

## 3. Results

### 3.1. The Characteristics of Four Groups in Patients with COPD

Table 1 presents the characteristics of the groups including pulmonary function and 6MWD. The LoFlo + HiEx, HiFlo + HiEx, LoFlo + LoEx, and HiFlo + LoEx groups contained 50, 78, 69, and 69 subjects, respectively. The HiFlo + HiEx group was a low value intentionally as compared with other groups. The LoFlo + HiEx and LoFlo + LoEx groups had a low %FEV_1.0_, but the LoFlo + HiEx group had a significantly higher %FVC (*p* < 0.01).

The LoFlo + HiEx group demonstrated a significantly better %MEP (*p* < 0.01) and %MIP (*p* < 0.05, *p* < 0.01) than the LoFlo + LoEx and HiFlo + LoEx groups, as well as better grip power, %KE, MGS, TUG, CS-30, ISWD, and NRADL (*p* < 0.01). The LoFlo + HiEx group did not demonstrate significant differences in any of these indicators when compared with the HiFlo + HiEx group, which also had high exercise tolerance. Compared with the HiFlo + HiEx group, the HiFlo + LoEx group had a significantly poorer %MEP, %MIP, grip power, %KE, MGS, TUG, CS-30, and ISWD (*p* < 0.01). There were no significant differences in these indicators between the HiFlo + LoEx and LoFlo + LoEx groups, both of which demonstrated low exercise tolerance. There were significant differences in mMRC scores between all groups; the HiFlo + HiEx group had the best score, followed by the LoFlo + HiEx and HiFlo + LoEx groups, and the LoFlo + LoEx group had the poorest score. The LoFlo + HiEx and HiFlo + HiEx groups demonstrated high exercise tolerance, but the LoFlo + HiEx group had a significantly poorer mMRC score (*p* < 0.01). The HiFlo + LoEx and LoFlo + LoEx groups demonstrated low exercise tolerance, but the HiFlo + LoEx group had a significantly poorer mMRC score (*p* < 0.05).

The HiFlo + HiEx group had the best NRADL score, followed by the LoFlo + HiEx and HiFlo + LoEx groups, and the LoFlo + LoEx group had the poorest score. Significant differences were observed between all pairs of groups, except for the LoFlo + HiEx and HiFlo + HiEx groups. There was no significant difference in SGRQ scores between the LoFlo + HiEx and HiFlo + HiEx groups, both of which had high exercise tolerance; however, comparison of the HiFlo + LoEx and LoFlo + LoEx groups, both of which had low exercise tolerance, revealed a significantly better score in the HiFlo + LoEx group (*p* < 0.01). For %IBW and HADS (*n* = 86), the LoFlo + HiEx and HiFlo + LoEx groups did not demonstrate significant differences when compared with any other group.

### 3.2. Relationship with Social Background in the LoFlo + HiEx and HiFlo + LoEx Groups

To clarify factors that could affect their exercise tolerance, we compared the LoFlo + HiEx and HiFlo + LoEx groups. More participants in the LoFlo + HiEx group were driving a car (χ^2^ = 10.89, *p* < 0.05), fewer participants in the HiFlo + LoEx group did not exercise (χ^2^ = 7.79, *p* = 0.05), and more participants in the HiFlo + LoEx group had not been hospitalized in the previous year for respiratory illness (χ^2^ = 10.97, *p* < 0.05; Table 2).

### 3.3. Comparison of Rehabilitation Effects in the LoFlo + HiEx and HiFlo + LoEx Groups

**Figure 3:**
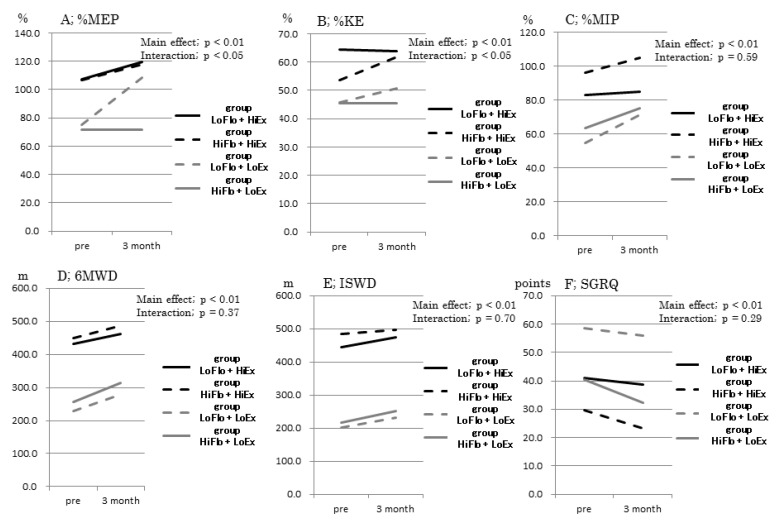
Comparison of the pulmonary rehabilitation effect for three months between each group. %KE, ratio of knee extension strength to body weight; %MEP, ratio of maximal expiratory pressure to body weight; %MIP, ratio of maximal inspiratory pressure to body weight; 6MWD, six-minute walking distance; ISWD, incremental shuttle walking distance; SGRQ, St. George’s Respiratory Questionnaire.

The ANOVA with a two-factor spilt-plot design revealed that %MEP (*p* < 0.01, *p* < 0.05) and %KE (*p* < 0.05, *p* < 0.05) had a significant main effect and interaction. However, although %MIP (*p* < 0.05), MGS, TUG, 6MWD, ISWT, SGRQ (*p* < 0.01) had a significant main effect, there was no interaction (Figure 3; A~F; Table S1).

The LoFlo + HiEx group demonstrated significantly less change in %KE than the LoFlo + LoEx group (*n* = 24; *p* < 0.05), while the HiFlo + LoEx group demonstrated significantly greater change in ISWD than the HiFlo + HiEx group (*n* = 30, *p* < 0.05). The LoFlo + HiEx and HiFlo + LoEx groups did not demonstrate significant between-group differences in rates of change for any other indicators (Table 3).

## 4. Discussion

In analysis 1, we conducted a cross-sectional investigation of respiratory function, physical function, and physical ability in the LoFlo + HiEx and HiFlo + LoEx groups at baseline. Our results suggest that younger participants in the LoFlo + HiEx group, even with low respiratory function, demonstrated higher exercise tolerance. The LoFlo + HiEx group had the same high exercise tolerance as the HiFlo + HiEx group, as well as high respiratory function, physical function, and physical ability. Seymour et al. [15] reported that the percentage of COPD patients with muscle atrophy increases with advancing GOLD stage. However, in this study, participants in the LoFlo + HiEx group, who would be classified as GOLD stage 3 or 4 had the respiratory muscle and quadriceps strength of participants with high exercise tolerance; thus, participants of the LoFlo + HiEx group could maintain a level of physical ability in terms of MGS, TUG, CS-30, and IWSD equivalent to that of participants with high exercise tolerance.

However, the HiFlo + LoEx group, which had favorable respiratory function (%FEV_1.0_), performed poorer than the LoFlo + HiEx group on several indicators of physical function and ability. Furthermore, the HiFlo + LoEx group who, based on GOLD stage (%FEV_1.0_) would be assumed to have the same physical function and ability as those with high exercise tolerance, had low exercise tolerance, which resulted in an early decline in physical function (e.g., respiratory muscle and quadriceps strength) and ability (e.g., MGS, TUG, CS-30, and ISWD). Thus, the HiFlo + LoEx group demonstrated high physical function and ability despite being in a severe airway obstruction; conversely, the HiFlo + LoEx group demonstrated low physical function and ability despite being in a mild airway obstruction of COPD.

Prior studies have reported that reduced skeletal muscle and gait function in patients with COPD depends on deconditioning [16], age-related sarcopenia [17], and nutritional status [18]. The present study has yielded the novel finding that inconsistent low exercise tolerance also promotes a reduction in skeletal muscle and gait function. Agusti et al. [19] reported that SGRQ scores gradually worsen with advancing GOLD stage (stage 2, 42.5 points; stage 3, 54.0 points; stage 4, 61.5 points). In the present study, the LoFlo + HiEx Group (GOLD stage 3–4) had an SGRQ score of 43.2, so they were able to maintain a QoL equivalent to that found in patients with GOLD stage 2. Meanwhile, the HiFlo + LoEx group had an SGRQ score of 41.7, so they were able to maintain a QoL equivalent to that of patients with GOLD stage 2 despite having GOLD stage 4 exercise tolerance [19]. However, many studies have reported that QoL and FEV_1.0_ are not correlated [20,21,22]. Several factors have been reported to impair QoL, including reduction in exercise tolerance and physical activity, because of obstructive respiratory disorders, gas exchange disorders, malnutrition, and skeletal muscle dysfunction [23]. The present study demonstrated that patients with COPD who present with various exercise tolerance experience less impairment to QoL.

Dyspnea was more severe in the LoFlo + HiEx group than in those with high exercise tolerance, while dyspnea was milder in the HiFlo + LoEx group than in those with low exercise tolerance. This observation suggests that varying exercise tolerance causes patients with high exercise tolerance to experience more severe dyspnea and those with low exercise tolerance to experience less severe dyspnea. In addition, the finding that the LoFlo + HiEx group had milder dyspnea than those in the HiFlo + LoEx group suggests that dyspnea may depend more on exercise tolerance than on degree of airflow obstruction (i.e., %FEV_1.0_, GOLD stage).

The finding that the HiFlo + LoEx group performed better in ADL than those with low exercise tolerance demonstrated that among patients with COPD with low exercise tolerance, presentation of various exercises of differing difficulties led to better performance of ADL. However, among patients with high exercise tolerance, presentation of various exercises of differing difficulties did not yield a difference in ADL. No relationship was found between exercise tolerance and %IBW or scores on the HADS and its subscales.

In analysis 2, it was found that large number of participants in the LoFlo + HiEx group could drive a car, whereas few in the HiFlo + LoEx group did so. For patients with COPD, driving a car is an important way of expanding their living space. Our findings indicate that driving has the potential to convert patients with advanced COPD (i.e., reduced %FEV_1.0_) into the LoFlo + HiEx group. In contrast, the small number of the HiFlo + LoEx group who were driving indicates that narrowing of the living space has the potential to create the HiFlo + LoEx group. A lack of exercise has the potential to create the HiFlo + LoEx group, even if they are in the early stage of COPD.

Analysis 3 was a longitudinal investigation of the effects of the three-month pulmonary rehabilitation program in participants of the LoFlo + HiEx or HiFlo + LoEx groups, in terms of changes in indicators of respiratory function and physical function or ability.

Following rehabilitation, the LoFlo + HiEx group demonstrated significant improvements in dyspnea, walking speed, and exercise tolerance, while the HiFlo + LoEx group demonstrated significant improvements in respiratory muscle strength, walking speed, exercise tolerance, and QoL.

Between-group comparison of rates of change showed that patients with COPD presenting with varying exercise tolerance did not show any particular differences in the short-term effects of pulmonary rehabilitation. However, lower limbs muscular power was more difficult to improve in the LoFlo + HiEx and HiFlo + LoEx groups compared with the HiFlo + HiEx and LoFlo + LoEx groups.

A number of studies have reported that the effects of pulmonary rehabilitation are greatest in patients with moderate clinical symptoms [24,25]. Although our consideration is hypothetical, this finding may reflect the fact that patients with moderate symptoms are more motivated than those with mild symptoms while also tolerating exercise better than those with severe symptoms. Walking ability and exercise tolerance may have been more easily improved in the HiFlo + LoEx group with early COPD. However, the lack of improvement in skeletal muscle strength in the extremities in the LoFlo + HiEx and HiFlo + LoEx groups suggests involvement of factors other than respiratory function.

Based on previous data, the effects of pulmonary rehabilitation on instantaneous abilities were less likely to be found in the HiFlo + LoEx group, whereas the effects of pulmonary rehabilitation on aerobic capacity was more likely to occur in all groups, including the HiFlo + LoEx group. Dyspnea improved significantly in the LoFlo + HiEx group and in those with low exercise tolerance (both groups with poorer respiratory function). However, there was little improvement in dyspnea in the HiFlo + LoEx group or in those with high exercise tolerance (both groups with better respiratory function). In contrast, although QoL improved significantly in the HiFlo + LoEx group and those with high exercise tolerance, improvement in QoL was poor in the LoFlo + HiEx group and those with low exercise tolerance (both groups with poorer respiratory function). These results were presumed to be due to dependence of dyspnea on the degree of lung hyperinflation, and the fact that COPD patients with impaired respiratory function (i.e., patients with considerable airway obstruction) were more likely to be affected. This is due to the “individual growth in competence” developing in patients with dyspnea because they had the opportunity to undergo pulmonary rehabilitation. Meanwhile, various factors can be involved in the QOL, due to the fact that “individual growth in competence” was greater in patients with less advanced stage of disease who had good respiratory function.

Although no studies have examined the minimal clinically important difference (MCID) for the 6MWD in Japanese subjects, previous studies have reported figures of 25 m [26] and 35 m [27]. A further study, which used data from part of the ECLIPSE study, reported that a reduction in MCID of 30 m in one year increased the risks of hospitalization and death [28]. In the present study, all groups demonstrated an improvement in 6MWD that was greater than the MCID. In addition, the HiFlo + LoEx group demonstrated a significantly higher rate of change in ISWT than those with high exercise tolerance, suggesting that patients with low exercise tolerance and adequately maintained respiratory function can easily improve their exercise tolerance.

The limitation of this study was that the outpatient respiratory rehabilitation system could not be constructed. Hence, except for the 89 participants, the analysis was limited to the initial data, and longitudinal studies could only track the progress for three months. In this study, the exercise tolerance of the subjects in the HiFlo + LoEx group was the most improved, but there is a concern that the HiFlo + LoEx group may not necessarily benefit from respiratory rehabilitation over the long term. Moving forward, we want to observe the progression of these four COPD patient categories over the long term.

## 5. Conclusions

We suggest that the HiFlo + LoEx group had a greatest effect of three months of pulmonary rehabilitation compared to all other groups studied. COPD patients with low exercise tolerance despite mild airflow obstruction may benefit from pulmonary rehabilitation.

The GOLD Workshop Report calls for more individualized medical care at the level of the individual patient. In the present study, we divided patients based on GOLD stage and exercise tolerance, thereby proposing novel categories for provision of individualized medical care. Further investigations that can extend our findings are needed to guide further individualized medical care for patients with COPD.

### 5.1. What is Already Known on This Topic

In the Combined COPD Assessment (the refined ABCD assessment tool) using GOLD, COPD is categorized by acute exacerbation and the mMRC or COPD Assessment Test score. Many previous studies have compared the characteristics of physical ability and training effects by category. In recent years, the GOLD stage does not reflect the severity of COPD but has been considered as an indicator of airway obstruction.

### 5.2. What This Study Adds

Rather than dyspnea, this study was categorized according to the more objective exercise tolerance test (6MWD), which is strongly related to dyspnea, and according to the respiratory function, which is the most common clinical indicator. Following this, the study compared the characteristics of physical ability and the effects of training. Due to the lack of previous studies on this topic, the comparison of these categories can be clinically useful indicators.

## Figures and Tables

**Table 1:** Comparison of the characteristics of the LoFlo + HiEx, HiFlo + HiEx, LoFlo + LoEx, and HiFlo + LoEx groups.

	LoFlo + HiEx Group	HiFlo + HiEx Group	LoFlo + LoEx Group	HiFlo + LoEx Group	
*n*	50	78	69	38	
male, %	96	87.2	92.8	94.7	
age, years old	69.1 ± 10.0	71.9 ± 7.3	75.7 ± 7.8	79.2 ± 7.3	^††, ‡‡^
%FEV_1.0_, %	36.0 ± 7.9	74.5 ± 16.8	33.2 ± 9.6	74.4 ± 17.6	^**, ‡‡, ||||^
6MWD, m	433 ± 59	456 ± 71	225 ± 99	252 ± 68	^††, ‡‡, §§^
%FVC, %	72.7 ± 19.3	91.7 ± 16.1	59.4 ± 16.1	86.1 ± 21.9	^**, ††, ‡‡, ||||^
%MEP, %	96.5 ± 35.7	89.0 ± 32.9	65.0 ± 32.0	65.0 ± 32.2	^††, ‡‡, §§^
%MIP, %	95.4 ± 41.3	90.9 ± 35.2	71.3 ± 40.5	67.0 ± 31.8	^†, ‡‡, §^
%IBW, %	99.1 ± 18.9	100.9 ± 17.1	91.1 ± 18.9	97.2 ± 15.9	
mMRC	1.7 ± 0.8	1.2 ± 0.7	2.8 ± 0.9	2.2 ± 0.8	^**, ††, ‡, §§, ||^
GP, kg	34.2 ± 7.6	30.2 ± 8.3	25.2 ± 7.6	24.8 ± 7.4	^*, ††, ‡‡, §§^
%KE, %	60.5 ± 15.3	55.3 ± 14.2	44.2 ± 14.3	45.1 ± 10.7	^††, ‡‡, §§^
MGS, m/min	113.6 ± 20.3	114.0 ± 22.3	81.0 ± 27.1	86.1 ± 18.0	^††, ‡‡, §§^
TUG, sec	5.9 ± 0.9	6.1 ± 1.3	9.7 ± 5.2	9.5 ± 4.0	^††, ‡‡, §§^
CS-30, times	18.0 ± 4.2	18.2 ± 4.3	12.0 ± 4.7	12.6 ± 3.3	^††, ‡‡, §§^
ISWD, m	404 ± 125	446 ± 156	193 ± 90	197 ± 95	^††, ‡‡, §§^
NRADA, points	80.5 ± 13.8	88.5 ± 12.9	54.7 ± 22.7	67.0 ± 20.5	^††, ‡‡, §§, ||||^
SGRQ points	43.2 ± 17.1	34.9 ±1 7.5	55.8 ± 17.0	41.7 ± 13.3	^††, ||||^
HADS(A), points (*n* = 86)	6.2 ± 2.8	4.4 ± 3.0	6.0 ± 3.6	5.7 ± 3.9	
HADS(D), points (*n* = 86)	7.9 ± 3.4	5.8 ± 3.0	8.2 ± 3.7	7.4 ± 2.9	

Values are mean ± SD. *: LoFlo + HiEx vs. HiFlo + HiEx, *p* < 0.05, **: *p* < 0.01. †: LoFlo + HiEx vs. LoFlo + LoEx, *p* < 0.05, ††: *p* < 0.01. ‡: LoFlo + HiEx vs. HiFlo + LoEx, *p* < 0.05, ‡‡: *p* < 0.01. §: HiFlo + HiEx vs. HiFlo + LoEx, *p* < 0.05, §§: *p* < 0.01. ||: LoFlo + LoEx vs. HiFlo + LoEx, *p* < 0.05, || ||: *p* < 0.01. %FEV_1.0_, percentage of predicted forced expiratory volume in 1 s; %FVC, percentage of predicted forced vital capacity; %IBW, body weight divided by standard body weight; %KE, ratio of knee extension strength to body weight; %MEP, ratio of maximal expiratory pressure to body weight; %MIP, ratio of maximal inspiratory pressure to body weight; 6MWD, six-minute walking distance; CS-30, 30-s chair stand times; GP, grip power; HADS (A), Hospital Anxiety and Depression Scale (Anxiety); HADS (D), Hospital Anxiety and Depression Scale (Depression); ISWD, incremental shuttle walking distance; MGS, maximum gait speed; mMRC, modified Medical Research Council dyspnea scale; NRADL, Nagasaki University Respiratory ADL questionnaire; SGRQ, St. George’s Respiratory Questionnaire; TUG, timed up and go time.

**Table 2:** Relationship with social background in the LoFlo + HiEx, HiFlo + HiEx, LoFlo + LoEx and HiFlo + LoEx groups.

	χ^2^ Value	*p* Value
Employment status (employed or unemployed)	5.04	0.54
Marital status (married or not married)	5.64	0.13
Alcohol consumption (yes or no)	10.59	0.01
Smoking (yes or no)	2.19	0.53
Exercise habits (yes or no)	7.79	0.05
Driving a car (yes or no),	10.89	0.01
Comorbidities: cancer (yes or no)	7.22	0.07
Comorbidities: heart disease (yes or no)	6.51	0.09
Comorbidities: hyperlipidemia (yes or no)	2.29	0.52
Comorbidities: hypertension (yes or no)	3.12	0.38
Comorbidities: diabetes mellitus (yes or no)	1.64	0.65
Home oxygen therapy (yes or no)	26.01	<0.01
House structure (one-story or two-story)	2.59	0.46
Environment around the home (flat or hilly)	9.01	0.03
Hospitalization for respiratory illness within the past year (yes or no)	10.97	0.01
Exacerbation within the past year (yes or no)	7.27	0.06

**Table 3:** Comparison of the rate of change between LoFlo + HiEx, HiFlo + HiEx, LoFlo + LoEx, and HiFlo + LoEx groups.

	LoFlo + HiEx Group (*n* = 19)	HiFlo + HiEx Group (*n* = 30)	LoFlo + LoEx Group (*n* = 24)	HiFlo + LoEx Group (*n* = 16)	
%MEP	14.8 ± 17.9	14.7 ± 24.8	59.5 ± 78.4	35.9 ± 48.5	^§^
%MIP	9.0 ± 44.9	11.2 ± 29.9	33.9 ± 62.3	26.2 ± 39.1	
%IBW	−0.2 ± 2.2	−1.8 ± 6.9	−0.5 ± 4.5	4.3 ± 19.2	
mMRC	−32.0 ± 40.7	−16.7 ± 42.2	−4.2 ± 29.3	−17.8 ± 23.1	
GP	2.1 ± 13.3	4.4 ± 17.2	7.8 ± 23.2	1.9 ± 8.9	
%KE	−1.1 ± 16.9	18.8 ± 29.4	22.4 ± 33.4	1.6 ± 13.9	^†^
MGS	11.1 ± 16.5	3.9 ± 9.3	24.9 ± 47.1	15.9 ± 15.7	
TUG	−5.9 ± 10.9	−6.8 ± 9.2	−9.2 ± 26.9	−11.5 ± 9.5	
CS-30	6.6 ± 18.9	12.1 ± 18.3	8.7 ± 11.5	13.8 ± 12.2	
6MWD	7.7 ± 16.2	8.0 ± 8.6	31.0 ± 47.1	27.6 ± 31.2	^§^
ISWD	7.3 ± 14.4	4.2 ± 13.7	20.9 ± 27.3	23.1 ± 30.8	^#^
NRADL	−0.2 ± 15.4	2.7 ± 6.2	8.6 ± 22.7	1.0 ± 27.8	
SGRQ	0.5 ± 33.2	−17.7 ± 34.6	−2.8 ± 20.7	−20.2 ± 24.9	

Values are mean ± SD. ^†^: LoFlo + HiEx vs. LoFlo + LoEx, *p* < 0.05. ^#^: HiFlo + HiEx vs. HiFlo + LoEx, *p* < 0.05. ^§^: HiFlo + HiEx vs. LoFlo + LoEx, *p* < 0.05. %IBW, body weight divided by standard body weight; %KE, ratio of knee extension strength to body weight; %MEP, ratio of maximal expiratory pressure to body weight; %MIP, ratio of maximal inspiratory pressure to body weight; 6MWD, six-minute walking distance; CS-30, 30-s chair stand times; GP, grip power; ISWD, incremental shuttle walking distance; MGS, maximum gait speed; mMRC, modified Medical Research Council dyspnea scale; NRADL, Nagasaki University Respiratory ADL questionnaire; SGRQ, St. George’s Respiratory Questionnaire; TUG, timed up and go time.

## Data Availability

Not applicable.

## Supplementary Materials

The following are available online at https://www.mdpi.com/2227-9032/9/1/53/s1, Table S1: Comparison of pulmonary rehabilitation effects between the LoFlo + HiEx, HiFlo + HiEx, LoFlo + LoEx, and HiFlo + LoEx groups.
