# Supplementary material for: Validation of Clinical Characteristics and Effectiveness of Pulmonary Rehabilitation in a COPD Population with Discrepancy between Exercise Tolerance and FEV1
Source: Healthcare (Basel). 2021 Jan 6;9(1):53. doi: 10.3390/healthcare9010053 (PMC7825294; doi:10.3390/healthcare9010053)
Supplement: Supplementary file 1 [file healthcare-09-00053-s001.pdf]

Table S1

Comparison of pulmonary rehabilitation effects between the A, B, C, and D groups

	Group A (n=19)		Group B (n=30)		Group C (n=24)		Group D (n=16)		Comparison between groups	
	Baseline	3 month	Baseline	3 month	Baseline	3 month	Baseline	3 month	Baseline	3month
%MEP, %	107.0 ± 38.2	119.3 ± 38.0	106.9 ± 28.5	117.8 ± 22.9*	75.3 ± 32.2	108.2 ± 38.1**	71.9 ± 30.4	89.6 ± 26.8**	†, ‡, #	
%MIP, %	82.7 ± 36.9	84.7 ± 35.9	95.9 ± 28.3	104.7 ± 31.8	54.5 ± 22.1	71.2 ± 43.2*	63.4 ± 25.2	75.0 ± 25.4	†, ##	
%IBW, %	99.3 ± 19.4	98.9 ± 18.1	106.1 ± 13.8	104.0 ± 14.5	94.5 ± 17.7	94.1 ± 18.3	100.8 ± 15.5	103.9 ± 16.2		
mMRC	1.58 ± 0.90	1.05 ± 0.85**	1.10 ± 0.61	0.90 ± 0.61	2.82 ± 0.96	2.59 ± 0.96**	2.00 ± 0.85	1.53 ± 0.52	††, ##, ∯	††, ∯∯
GP, kg	34.3 ± 9.5	34.5 ± 7.8	29.1 ± 8.9	29.8 ± 8.5	23.7 ± 6.6	25.1 ± 6.9	25.1 ± 7.8	25.7 ± 8.5	††, ‡‡	††, ‡‡
%KE, %	64.3 ± 12.7	63.7 ± 17.8	53.5 ± 14.4	61.7 ± 14.3**	45.7 ± 12.6	50.8 ± 15.6*	45.5 ± 9.6	45.5 ± 7.6	¶¶, ††, ‡‡	†, ‡‡, ##
MGS, m/min	112.1 ± 16.2	123.5 ± 17.8*	118.6 ± 19.8	123.5 ± 23.6	83.8 ± 25.4	97.3 ± 18.9**	82.6 ± 13.0	94.5 ± 10.3*	††, ‡, ##	†, ‡, ##
TUG, sec	5.8 ± 0.7	5.4 ± 0.9	6.1 ± 1.1	5.7 ± 1.0	9.4 ± 5.7	7.4 ± 2.1**	8.4 ± 1.9	7.3 ± 1.3	††	††, ‡‡, ##
CS-30, times	17.5 ± 4.6	18.2 ± 4.1	18.0 ± 3.5	19.8 ± 3.3**	12.5 ± 3.0	13.5 ± 3.4	14.7 ± 4.3	16.3 ± 3.8	††	††
6MWD, m	432.3 ± 62.6	462.7 ± 80.4*	450.0 ± 64.7	486.9 ± 83.8**	227.8 ± 104.5	279.5 ± 119.6**	256.1 ± 73.3	312.8 ± 91.6**	††, ‡‡, ##	††, ‡‡, ##
ISWD, m	445.3 ± 153.5	473.5 ± 160.3	483.6 ± 165.3	497.5 ± 161.9	201.7 ± 93.1	232.8 ± 96.7*	215.7 ± 98.3	250.7 ± 95.4*	††, ‡‡, ##	††, ‡‡, ##
NRADL, points	81.4 ± 13.0	76.5 ± 24.6	90.9 ± 11.2	89.5 ± 19.3	58.4 ± 22.3	61.8 ± 21.8	75.1 ± 12.7	76.2 ± 23.2	††, ##, ∯∯	
SGRQ, points	40.9 ± 17.6	38.6 ± 16.6	29.7 ± 15.9	23.3 ± 14.9**	58.5 ± 18.5	56.0 ± 19.4	40.4 ± 12.7	32.4 ± 13.5**	†, ∯	†, ∯∯

Values are mean ± SD.

*: Baseline vs 3 month, $p < 0.05$; **: $p < 0.01$

¶: A vs B, p<0.05; ¶¶: p<0.01

†: A vs C, p<0.05; ††: p<0.01

‡: A vs D, p<0.05; ‡‡: p<0.01

#: B vs D, p<0.05; ##: p<0.01

∯: C vs D, p<0.05; ∯∯: p<0.01

%IBW, body weight divided by standard body weight; %KE, ratio of knee extension strength to body weight; %MEP, ratio of maximal expiratory pressure to body weight; %MIP, ratio of maximal inspiratory pressure to body weight; 6MWD, six-minute walking distance; CS-30, 30-second chair stand times; GP, grip power; ISWD, incremental shuttle walking distance; MGS, maximum gait speed; mMRC, modified Medical Research Council dyspnea scale; NRADL, Nagasaki University Respiratory ADL questionnaire; SGRQ, St. George's Respiratory Questionnaire; TUG, timed up and go time.
